# Disparities in Gastrointestinal Cancer Incidence in Asian American, Native Hawaiian, and Other Pacific Islander Groups

**DOI:** 10.1016/j.gastha.2025.100653

**Published:** 2025-03-15

**Authors:** Vicki Tang, Cynthia W. Ko

**Affiliations:** 1 Department of Medicine, University of Washington, Seattle, Washington; 2 Division of Gastroenterology, University of Washington, Seattle, Washington

**Keywords:** Asian American, Native Hawaiian, and Pacific Islander, Colorectal Neoplasm/Epidemiology, Stomach Neoplasms/Epidemiology, Esophageal Neoplasms/Epidemiology, Pancreatic Neoplasms/Epidemiology, Hepatocellular Neoplasms/Epidemiology

## Abstract

**Background and Aims:**

The Asian American, Native Hawaiian, and Other Pacific Islander (AANHPI) population are heterogeneous in health risk factors, socioeconomic status, and health outcomes. Disaggregating AANHPI groups may reveal disparities in cancer incidence. The aim of this study was to examine patterns and trends in incidence of common gastrointestinal cancers in AANHPI groups compared to the non-Hispanic White (NHW) population.

**Methods:**

Using the detailed AANHPI Surveillance, Epidemiology, and End Results database, we retrospectively analyzed trends in incidence of colorectal adenocarcinoma (CRC), gastric adenocarcinoma (GC), hepatocellular carcinoma, pancreatic adenocarcinoma, and esophageal cancer from 1990 to 2014 using Surveillance, Epidemiology, and End Results∗Stat and Joinpoint software, compared to NHW. Additional analyses were stratified by age at diagnosis for CRC (<50 and ≥50 years of age), for cardia and noncardia GC, and in esophageal cancer, for squamous cell carcinoma compared to esophageal adenocarcinoma.

**Results:**

CRC incidence was comparable in Hawaiian, Japanese, and NHW groups, with differing trends in younger and older age groups. Hepatocellular carcinoma incidence was highest in Chinese and Southeast Asian groups, while GC incidence was high in Other Pacific Islander, Korean, and Japanese groups. There was less variability in pancreatic adenocarcinoma incidence between NHW and AANHPI groups. AANHPI groups had a higher incidence of esophageal squamous cell carcinoma but a lower incidence of esophageal adenocarcinoma compared to NHW.

**Conclusion:**

Examining incidence of common gastrointestinal cancers in disaggregated AANHPI groups reveals differences in incidence rates and disparate trends over time. Further studies are needed to elucidate the reasons for these differing trends and to evaluate whether efforts to reduce cancer risk factors and promote appropriate cancer screening in high-risk AANHPI groups are needed to reduce cancer disparities.

## Introduction

The Asian American, Native Hawaiian, and Other Pacific Islander (AANHPI) population is the fastest growing single racial group in the United States and accounts for 6.2% of the US population in the 2020 census.^1^ However, there is heterogeneity between different AANHPI groups in health outcomes, socioeconomic status, educational attainment, and immigration status that can be easily overlooked when these groups are combined into a single category.^2^^,^^3^ Cancer statistics for AANHPI populations are often aggregated because of inaccurate or lack of population estimates and smaller population groups. Nevertheless, there have been studies examining cancer incidence in disaggregated Asian American groups for different cancer sites such as lung, prostate, and breast, and colorectal.^4^^,^^5^ Gastrointestinal cancers are of particular interest due to their relatively high incidence and the availability of effective screening modalities for colorectal cancer (CRC), gastric cancer (GC), and hepatocellular carcinoma (HCC).^6^, ^7^, ^8^

Prior studies have shown variability in cancer incidence and mortality patterns in AANHPI as a whole compared to non-Hispanic White (NHW) populations, with overall lower CRC and pancreatic cancer (PC) incidence but higher GC and HCC incidence.^3^, ^4^, ^5^^,^^9^^,^^10^ Although incidence of esophageal cancer (EC) is low compared to other cancer types, Asian Americans have a higher incidence of esophageal squamous cell carcinoma (ESquamCC) and a lower incidence of esophageal adenocarcinoma (EAdenoC), while the opposite is true in the general US population.^11^^,^^12^ These trends have also been shown in AANHPI populations in Hawaii; however, little is known about EC incidence among Pacific Islander groups in the general US population.^13^

Widespread population-based CRC screening is currently implemented in the US, but screening adherence varies among AANHPI subgroups.^14^^,^^15^ However, widespread population-based screening is not currently recommended or implemented in the US for GC, HCC, PC, or EC. Targeted screening for these cancers is practiced in high-risk groups. The aim of this study was to examine trends in incidence of common gastrointestinal cancers in census AANHPI groups to identify and better characterize differences in incidence and disparities between AANHPI groups, compared to NHW. Given the documented increase in early onset CRC incidence, we also aimed to examine differences in CRC incidence before and after the recommended average risk screening age (age 50 during the study period). This descriptive epidemiological study may help to inform the need for targeted risk factor reduction and cancer screening in AANHPI groups.

## Methods

We used the National Cancer Institute’s Surveillance, Epidemiology, and End Results (SEER) Registry to conduct a descriptive retrospective analysis of incidence of common gastrointestinal cancers in detailed AANHPI groups compared to NHW.^16^ We used a SEER-specialized database with detailed information available on disaggregated AANHPI subpopulations. This resource only includes data for a 25-year period from January 1, 1990, to December 31, 2014, for 13 regional SEER cancer registries which spanned registries in California, Connecticut, Hawaii, Iowa, New Jersey, New Mexico, Utah, Atlanta, Detroit, and Seattle. The registries represent approximately 27.7% of the Asian population and 47.2% of the Pacific Islander population in the United States.^17^ The other widely used SEER databases only allow for evaluation of AANHPI as a whole. In our analysis, we included the SEER-defined NHW, Chinese, Japanese, Filipino, Hawaiian, Korean, and Asian Indian and Pakistani groups. In this SEER database, cancer incidence in Asian Indian and Pakistani groups was combined for the entire study period. Due to the limited number of cases for some cancer sites, we aggregated Vietnamese, Laotian, and Kampuchean (Cambodian) groups as “Southeast Asian” and Guamanian/Chamorro and Samoan groups as “Other Pacific Islander”. SEER∗Stat software was used to determine age-adjusted incidence rates per 100,000 population-at-risk using the Tiwari et al., 2006 modification and the annual percent change (APC) with 95% confidence intervals.^18^ Population estimates were calculated by SEER using interpolation between 1990 and 2000 census data to account for ability to select multiple races in 2000, but not 1990.^16^ For groups that had incidence rate of 0 during any year between 1990 and 2014, APC could not be calculated. Joinpoint software was used to create joinpoint regression models to further characterize the direction and magnitude of incidence trends. A maximum of 4 joinpoints were allowed in our analysis.

We examined incidence in the most common histologic subtypes for each cancer site (Table A1). In our analyses, CRC and PC included only adenocarcinoma, GC included adenocarcinoma further subcategorized as cardia and noncardia, HCC was hepatocellular cancer, and EC included adenocarcinoma and squamous cell carcinoma both aggregated and separately. We examined incidence rate and trends stratified by age <50 years or ≥50 years for CRC only due to availability of adequate case numbers. We dichotomized at age 50 because it is a common age for initiation of cancer screening and was the recommended age for initiation of average-risk CRC screening during the study period. Analysis of incidence trends dichotomized by age for other cancer sites was limited by low case counts. We did not stratify findings by sex. Due to use of a deidentified database, this analysis was exempt from human subjects review at the University of Washington.

## Results

### Colorectal Adenocarcinoma

**Figure 1:**
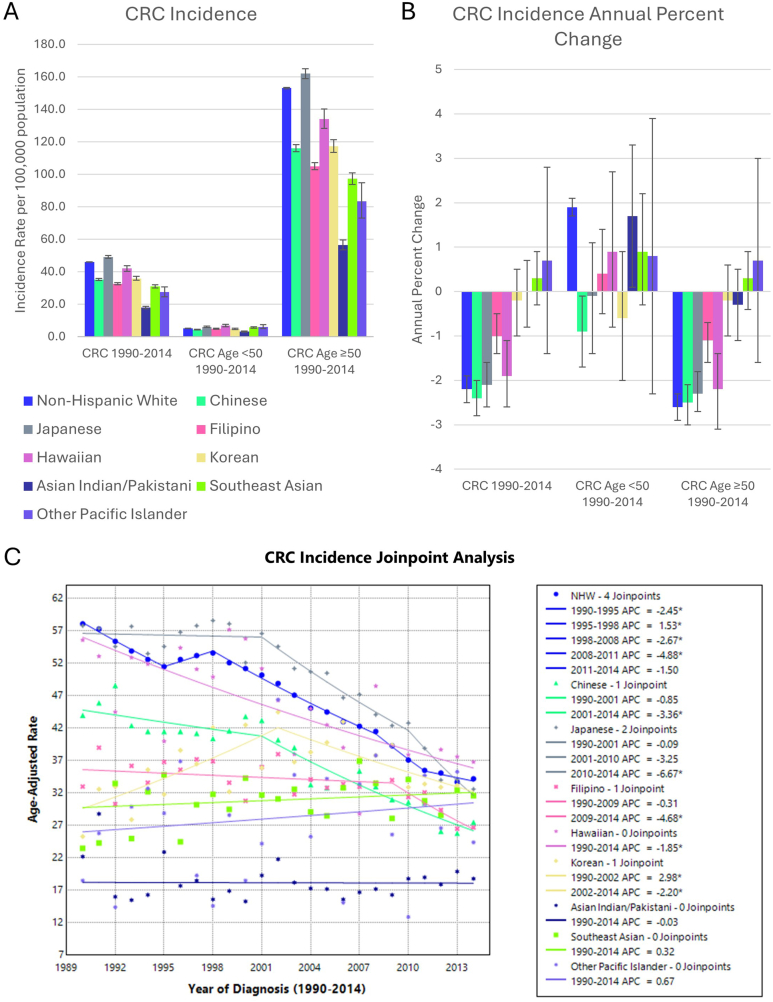
(A) CRC incidence per 100,000 population-at-risk, (B) APC, and (C) Joinpoint regression model in AANHPI groups. Error bars denote 95% confidence intervals. ∗ denotes statistical significance *P* < .05.

There were 519,223 CRC cases in NHW and 47,515 cases in combined AANHPI groups (Table 1). Asian Indian and Pakistani and Other Pacific Islander groups had the lowest CRC incidence (Figure 1). In contrast, CRC incidence was highest in Japanese and Hawaiian groups, similar to the incidence in NHW. CRC incidence decreased significantly over the study period in NHW, Chinese, Japanese, Filipino, and Hawaiian groups. Joinpoint analysis of overall incidence trends showed generally decreasing rates in NHW, with a period of increasing incidence from 1995 to 1998. In the Korean group, incidence increased from 1990 to 2002, then decreased from 2002 to 2014. In Chinese and Japanese groups, incidence started decreasing from 2001 to 2014 and in the Filipino group, incidence decreased from 2009 to 2014.

**Table 1:** Number of Cases of Each Gastrointestinal Cancer Type in AANHPI Groups

Cancer site	Number of cases									
	NHW	Combined AAPI	Chinese	Japanese	Filipino	Hawaiian	Korean	Asian Indian/Pakistani	Southeast Asian	Other Pacific Islander
Colorectal adenocarcinoma	519,223	47,515	12,007	11,596	10,627	2302	4234	2098	4240	411
Gastric adenocarcinoma	62,866	15,771	3551	4086	1775	582	3347	525	1682	223
Cardia	25,165	1738	394	484	322	82	170	130	133	23
Noncardia	37,701	14,033	3157	3602	1453	500	3177	395	1549	200
Hepatocellular carcinoma	38,656	15,344	4138	1648	2503	472	1945	481	3983	174
Pancreatic adenocarcinoma	90,255	8225	1790	1963	1837	491	829	446	774	95
Esophageal carcinoma	47,056	2869	700	754	420	215	212	310	229	29
Esophageal adenocarcinoma	30,840	614	122	158	160	48	16	80	30	<10
Esophageal squamous cell carcinoma	16,216	2248	578	596	260	167	196	230	199	22

AAPI, Asian American, Native Hawaiian, and Pacific Islander.

CRC incidence in individuals aged <50 was lower compared to those ≥50 years across all groups and years (Figure 1). In those age <50 years, CRC incidence increased in NHW and Asian Indian and Pakistani groups but decreased in the Chinese group. Joinpoint analysis showed the Japanese and Korean groups had increasing incidence from 1990 to 2005, then decreasing incidence from 2005 to 2014 in the age <50 years group (Figure A1). Similarly, in the Southeast Asian group, incidence increased from 1990 to 2000, then decreased from 2000 to 2005. In the ≥50 years group, CRC incidence decreased in NHW, Chinese, Japanese, Filipino, and Hawaiian groups but not in other AANHPI groups. Joinpoint analysis showed that the NHW and Korean groups had periods of increasing incidence from 1995 to 1998 and 1990–2002, respectively, mirroring the overall trends. In both Southeast Asian and Other Pacific Islander groups, there was a nonsignificant increase in CRC incidence in both the <50 years and the ≥50 years groups.

### Gastric Adenocarcinoma

**Figure 2:**
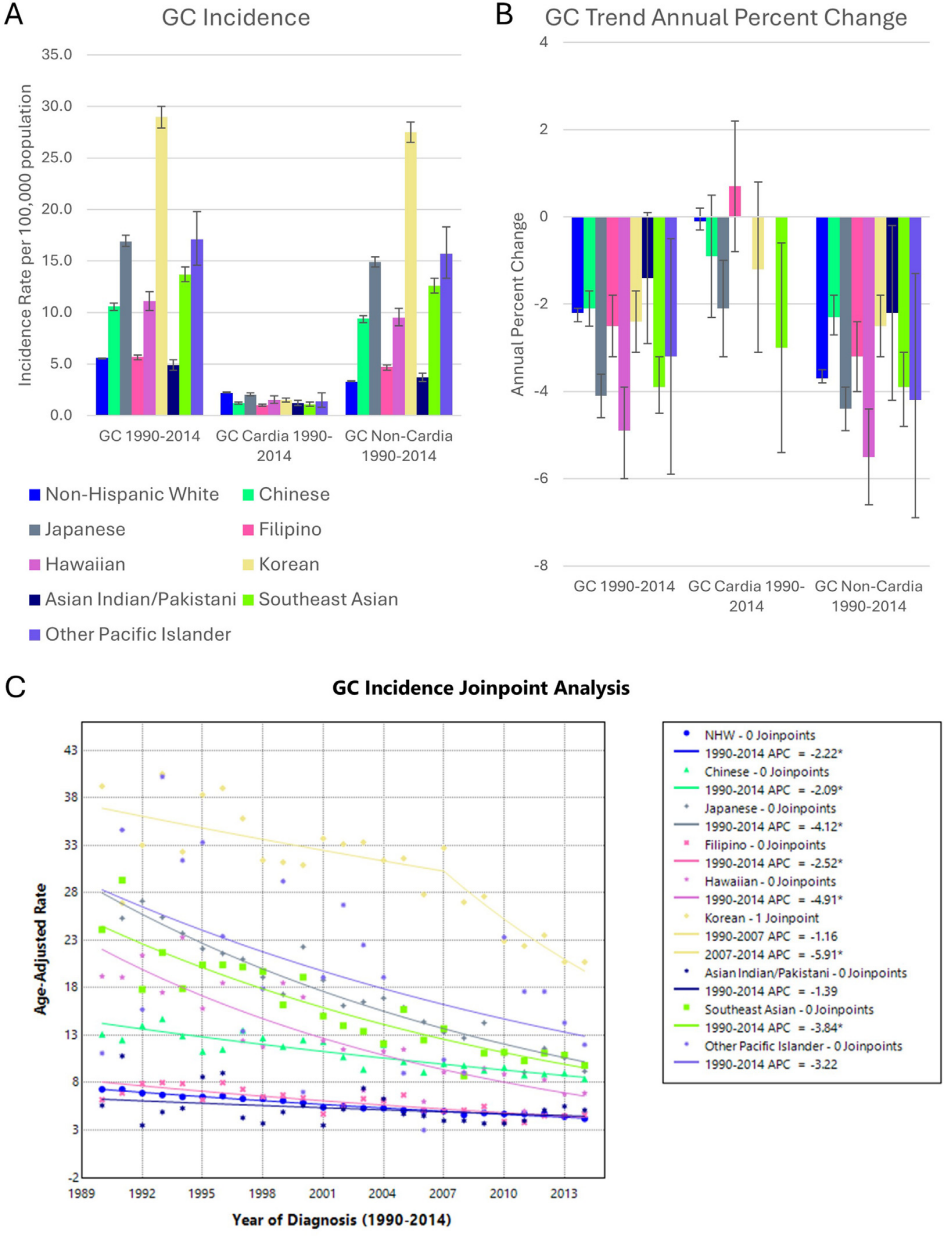
(A) GC incidence per 100,000 population-at-risk, (B) APC, and (C) Joinpoint regression model in AANHPI groups. Error bars denote 95% confidence intervals. ∗ denotes statistical significance *P* < .05.

There were 62,866 cases of GC in NHW with 25,165 cardia cases, and 37,701 noncardia cases. Among AANHPI groups, there were 15,771 GC cases with 1738 cardia cases and 14,033 noncardia cases (Table 1). Incidence of noncardia GC was higher than cardia GC in all AANHPI groups. Overall GC incidence closely mirrored noncardia GC incidence, which was highest in Korean, Japanese, and Other Pacific Islander groups and lowest in NHW, Filipino, and Asian Indian and Pakistani groups (Figure 2). Overall and noncardia GC incidence decreased significantly in all groups except the Asian Indian and Pakistani group, with the greatest decrease in the Hawaiian group (Figure A2). Cardia GC incidence decreased in Japanese and Southeast Asian groups.

### HCC

**Figure 3:**
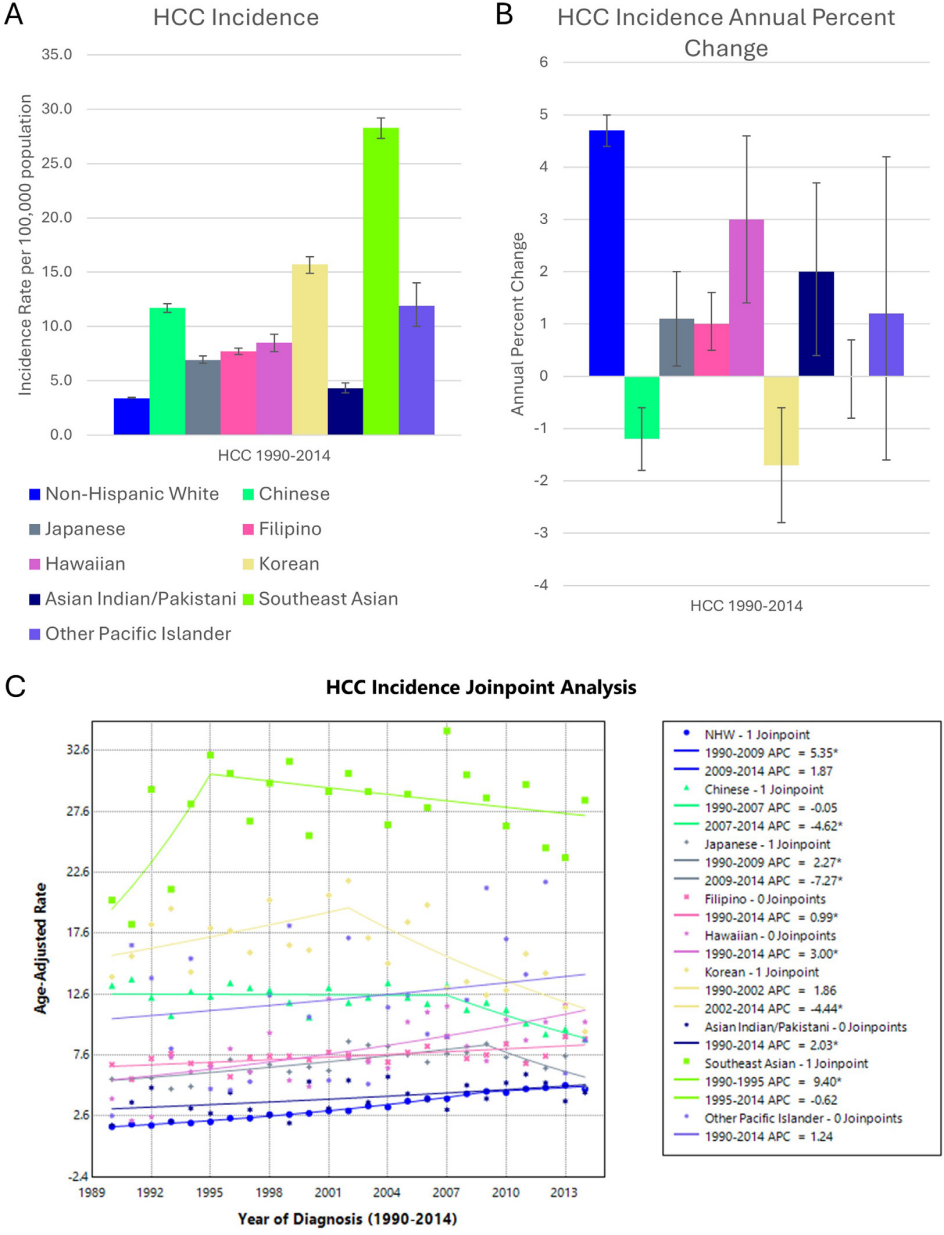
(A) HCC incidence per 100,000 population-at-risk, (B) APC, and (C) Joinpoint regression model in AANHPI groups. Error bars denote 95% confidence intervals. ∗ denotes statistical significance *P* < .05.

There were 38,656 cases of HCC in NHW, and 15,344 cases among AANHPI groups (Table 1). HCC incidence was highest in Southeast Asian, Korean, Chinese, and Other Pacific Islander groups and lowest in NHW and Asian Indian and Pakistani groups (Figure 3). HCC incidence increased significantly in NHW, Japanese, Filipino, Hawaiian, and Asian Indian and Pakistani groups, but decreased in Chinese and Korean groups. Joinpoint analysis showed that incidence increased in the Southeast Asian group from 1990 to 1995, before plateauing. Incidence increased in the Japanese group from 1990 to 2009, then decreased from 2009 to 2014. Similarly, incidence increased, though not statistically significantly, in the Korean group from 1990 to 2002 and then decreased from 2002 to 2014.

### Pancreatic Adenocarcinoma

**Figure 4:**
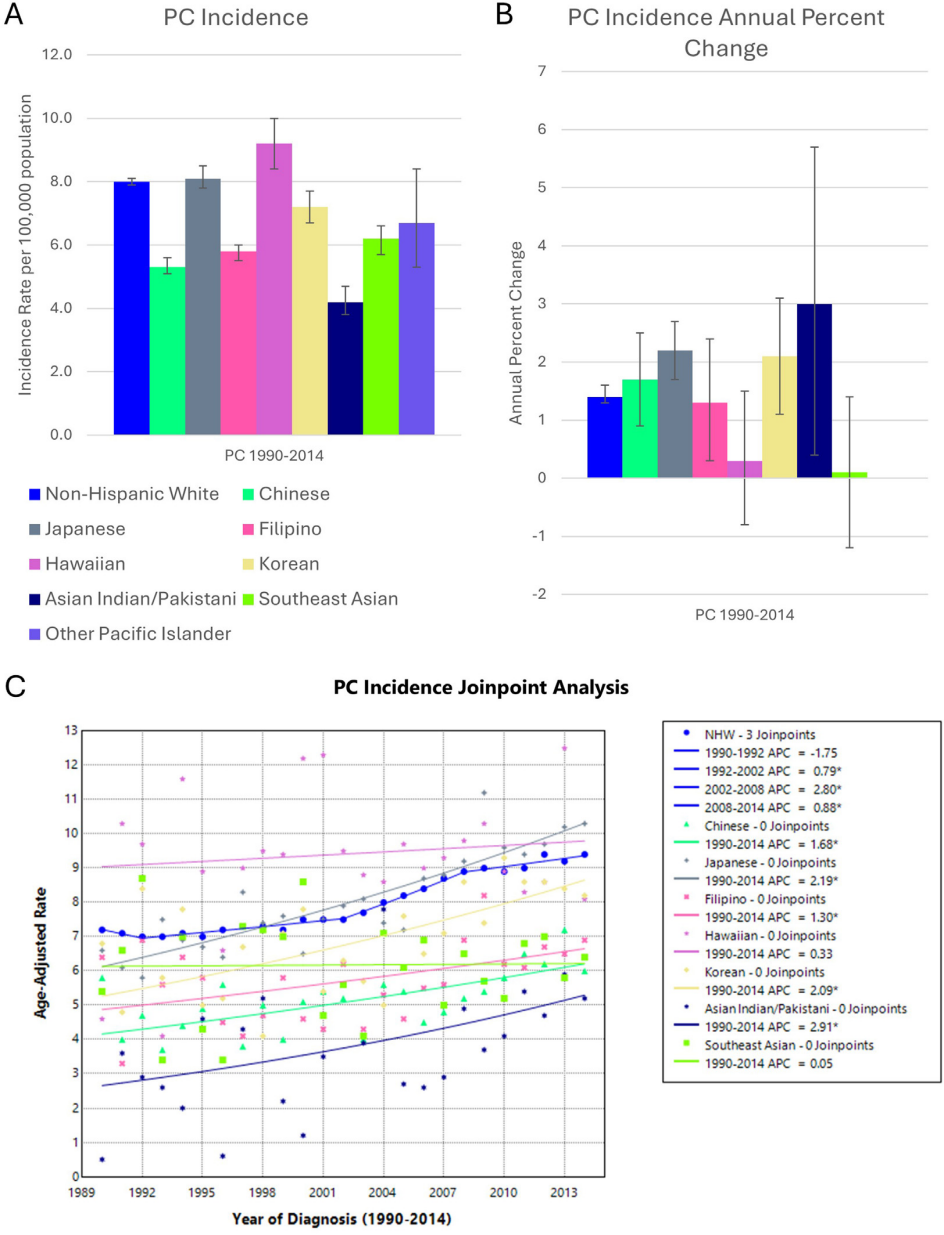
(A) PC incidence per 100,000 population-at-risk, (B) APC, and (C) Joinpoint regression model in AANHPI groups. Error bars denote 95% confidence intervals. ∗ denotes statistical significance *P* < .05.

There were 90,255 cases of PC in NHW, and 8255 cases in AANHPI groups (Table 1). In comparison to other gastrointestinal cancers, PC incidence varied less between groups, ranging between 4.2 and 9.2 per 100,000 population-at-risk (Figure 4). PC incidence increased in Chinese, Japanese, Filipino, Korean, and Asian Indian and Pakistani groups. Joinpoint analysis supported these trends.

### Esophageal Cancer

**Figure 5:**
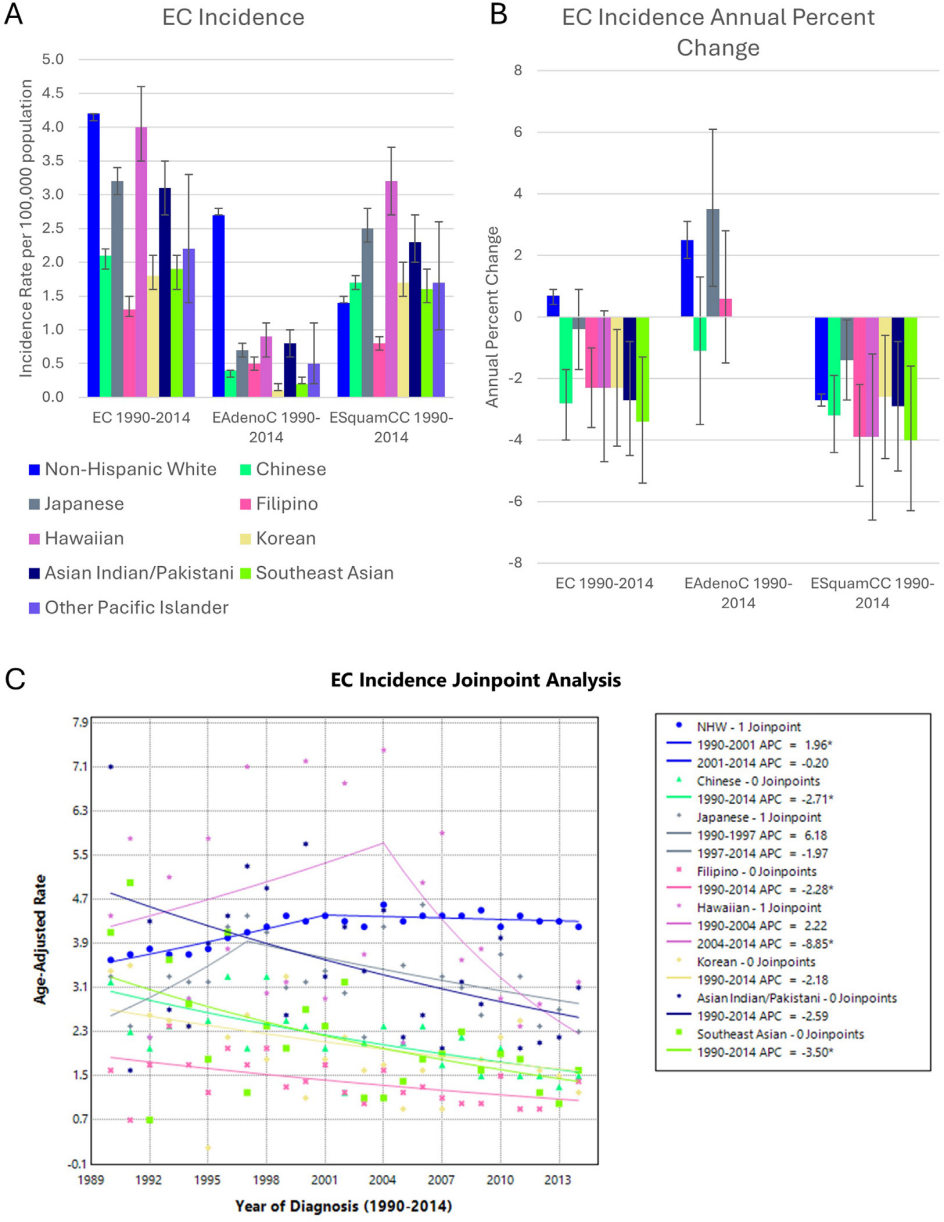
(A) EC incidence per 100,000 population-at-risk, (B) APC, and (C) Joinpoint regression model in AANHPI groups. Error bars denote 95% confidence intervals. ∗ denotes statistical significance *P* < .05.

There were 47,056 cases of EC in NHW, with 30,840 EAdenoC cases and 16,216 ESquamCC cases. In AANHPI, there were 2869 EC cases, with 621 EAdenoC cases and 2248 ESquamCC cases (Table 1). Overall EC incidence varied between 1.2 and 4.2 per 100,000 population-at-risk (Figure 5). NHW had a higher incidence of EAdenoC compared to ESquamCC; however, for all AANHPI groups, the incidence of ESquamCC was higher. ESquamCC incidence decreased in NHW and all AANHPI groups with sufficient data for analysis. EAdenoC incidence increased in NHW and Japanese groups, with no significant change in Chinese or Filipino groups. There were insufficient data for analysis of other AANHPI groups. In general, the overall EC incidence trend for the NHW group mirrored the EAdenoC incidence trend, while the overall EC incidence trends for AANHPI groups mirrored the ESquamCC incidence trend (Figure A3).

## Discussion

We identified substantial differences in gastrointestinal cancer incidence and trends in AANHPI groups from 1990 to 2014. Previous studies disaggregating AANHPI groups have also shown this heterogeneity. For example, CRC incidence increased in Korean, Filipino, and South Asian groups in a study of California from 1988 to 2007^19^; however, we found indeterminate overall trends in the latter two groups over our overlapping study period. While we found that CRC incidence was highest in Japanese and Filipino groups from 1990 to 2014, the American Cancer Society reports CRC is the most commonly diagnosed cancer in Korean, Hmong, and Cambodian Americans in 2023.^9^ Additionally, the trend of increasing incidence in NHW <50 years of age was not consistently seen in AANHPI groups, while the decreasing incidence in NHW age ≥50 was also not consistently seen in AANHPI groups. These variable trends may be due to differences in risk factor prevalence among different AANHPI groups compared to NHW. For example, there is variability in prevalence of obesity, tobacco use, alcohol use, and dietary risk factors across AANHPI groups and compared to NHW.^20^^,^^21^ In addition, factors that affect adherence to CRC screening including health literacy and attitudes, access to health insurance, and having a usual care provider also vary across AANHPI groups compared to NHW.^22^ Further studies are needed to better understand these evolving trends within AANHPI groups, which may inform potential development of interventions aimed at risk factor reduction or screening adherence to reduce CRC incidence disparities.

Most GC cases in AANHPI groups were noncardia which is correlated with *H. pylori* infection, a well-known risk factor in this population. Only a minority of cases were cardia in origin, which may be difficult to distinguish clinically from gastroesophageal junction malignancies arising from Barrett’s esophagus. While the high incidence of GC in Korean and Japanese Americans is well known,^23^^,^^24^ we also found high GC incidence in Southeast Asian and Other Pacific Islander groups, nearly comparable to Japanese Americans. A potential explanation for this variation in GC incidence includes differences in dietary intake of pickled foods and nitrites and nitrosamines through smoked foods in Korean and Japanese culture.^25^ Additionally, there is a high prevalence of *H. pylori* infection in Japan, Korean, and Southeast Asian countries^26^^,^^27^ which likely leads to higher prevalence in AANHPI immigrant groups. Despite the availability of both radiographic and endoscopic screening in GC, screening in the US relies on identification and targeted surveillance of high-risk individuals.^28^ In contrast, nationwide GC screening is implemented in Japan in adults ≥50 years and in South Korea in adults ≥40 years, and China has implemented an endoscopy-based screening program in certain geographic areas with high GC incidence.^29^ Further studies may build on these findings to determine if implementation of targeted risk factor assessment or screening of all AANHPI groups at increased risk of GC may be a strategy to address this disparity.

Disparities in HCC incidence have been shown within AANHPI groups.^30^ Our study showed HCC incidence was highest in the Southeast Asian group, nearly twice the rate of Koreans, the next highest group. Chronic hepatitis B virus (HBV) infection, a risk factor for HCC, disproportionately affects AANHPI, particularly those born in HBV endemic areas such as China and Southeast Asia.^31^^,^^32^ While prevalence of hepatitis C virus among AANHPI groups is generally lower, this may still vary among groups. For example, in one study in California hepatitis C virus was most prevalent in Cambodian and Vietnamese groups, but least prevalent in Chinese and Korean groups.^33^ Our findings suggest that evaluation for viral hepatitis, vaccination for HBV, and HCC screening should be targeted to populations with the highest risk of HCC such as Southeast Asians, which could lessen disparities between AANHPI groups. The previously mentioned differences in behavioral risk factors such as obesity, tobacco use, and alcohol use in AANHPI groups may also contribute to HCC incidence disparities.^20^^,^^21^^,^^34^

The small number of PC and EC cases among AANHPI limited our ability to analyze trends. In general, there was less overall variability in PC incidence. This may suggest fewer differences in the risk factors that drive PC such as tobacco use, alcohol use, and prevalence of chronic pancreatitis. Alternatively, it may indicate that such risk factors play a lesser role than genetic, environmental, or other predisposing factors.^35^ In contrast, we found interesting differences in EC according to the histologic subtype, with overall higher incidence of EAdenoC in NHW and higher incidence of ESquamCC in AANHPI groups. Differences in risk factors for the EC subtypes may underlie the variability in EAdenoC and ESquamCC rates.^12^^,^^13^ ESquamCC risk factors include tobacco and alcohol use, while risk factors for EAdenoC include Barrett’s esophagus, high body mass index, and gastroesophageal reflux disease.^11^^,^^20^ However, the increasing incidence of EAdenoC in the Japanese group, and to a lesser extent the Filipino group, may be indicative of changing lifestyle or assimilation of Western lifestyle or diet that place individuals at higher risk of EAdenoC. Targeted screening may be viable if these trends continue.

Additional factors that likely contributed to overall differences and disparities in cancer incidence include, but are not limited to, socioeconomic status, education attainment, cultural beliefs and health practices, immigration or migration, place of residence, and linguistic barriers.^15^^,^^30^^,^^36^^,^^37^ These factors not only affect adherence to guideline recommended screening for CRC, but may also affect timely diagnosis of cancers for which widespread population-based screening is not recommended in the US, such as GC, HCC, PC, or EC. For example, health literacy and recognition of common symptoms and risk factors of these cancers, as well as access to and trust of primary care physicians may be lower in AANHPI groups, particularly those with lower educational attainment and health literacy.^15^^,^^37^^,^^38^ AANHPI groups may also have less access to culturally sensitive health-care providers.^39^

There were limitations in our study. Due to the inherent limitations of cancer registry data, we did not have individual data on risk factors for the studied cancers or screening adherence, and could not assess how they influence cancer incidence and trends. This is especially limiting as some studies have suggested that cancer incidence, more so than mortality and survival is most closely linked to risk factors.^40^ Additional limitations are specific to the SEER database itself. Firstly, the datasets we used only included incidence data, and thus mortality and survival could not be assessed. Secondly, SEER registry data often have missing or incomplete data points such as unknown stage at diagnosis. Because of this, we did not include analyses stratified by stage at diagnosis. Third, race and ethnicity data are primarily derived from medical records and may be misclassified. Additionally, some combinations of groups and cancer sites had small case counts that limited our analyses. Fourth, while the SEER database encompasses a large portion of the US AANHPI population, due to limitations of statewide and regional databases, many geographical regions are not represented in our study. Lastly, data were only available from 1990 to 2014, and we could not assess how these trends have evolved since. While there are other SEER databases that encompass a larger geographical area and have more updated incidence data after 2014, our choice in SEER database was limited as this was the only database with disaggregated detailed AANHPI data. Fifth, there may be inaccuracies in categorization of cancers. For example, gastric cardia cancer is difficult to distinguish from distal esophageal cancer, as there may be anatomical and histological overlap. While prior documentation of Barrett’s esophagus may help distinguish between the 2 cancer sites, these data are not available in the SEER database.

In summary, we showed differences in incidence patterns and trends in CRC, GC, HCC, GC, and EC between AANHPI groups and compared to NHW. Continuing to disaggregate AANHPI groups in cancer databases can help to elucidate cancer disparities among AANHPI groups, and lead to more nuanced consideration for cancer control screening efforts. This variability also suggests the additional need to identify the underlying reasons for these disparities, which may inform development of targeted and culturally appropriate efforts toward risk factor reduction and appropriate cancer screening.

## Figures and Tables

**Supplementary Figure:**
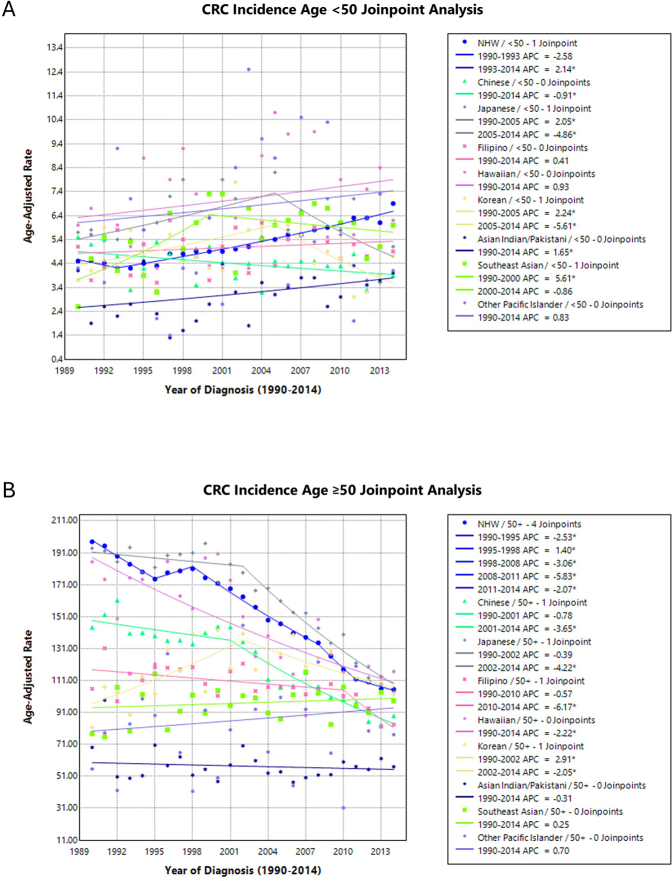

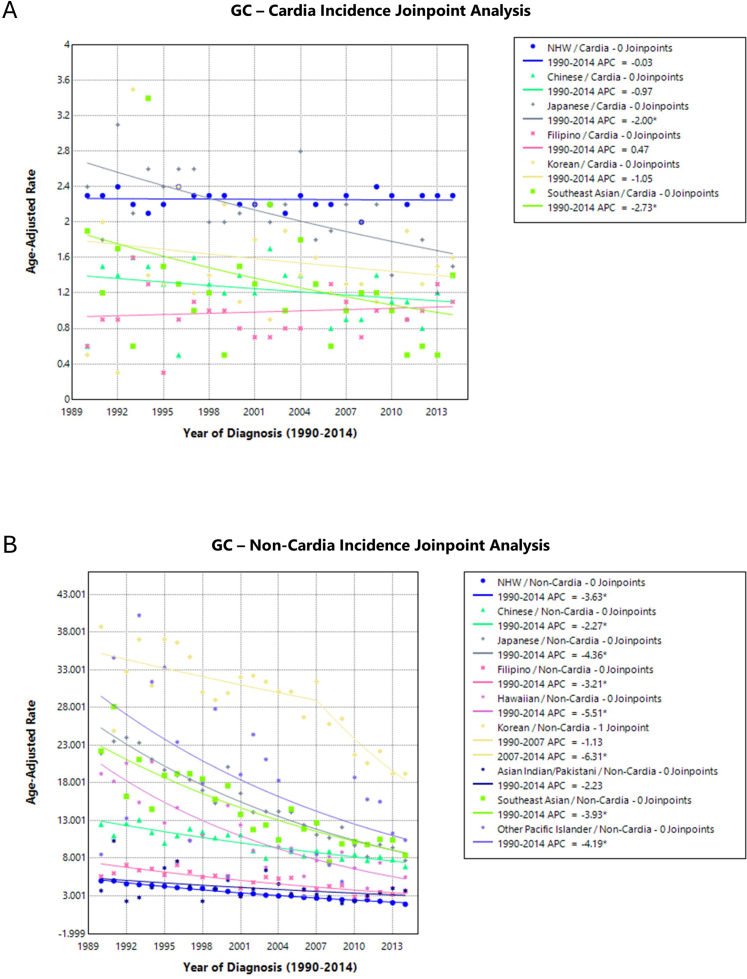

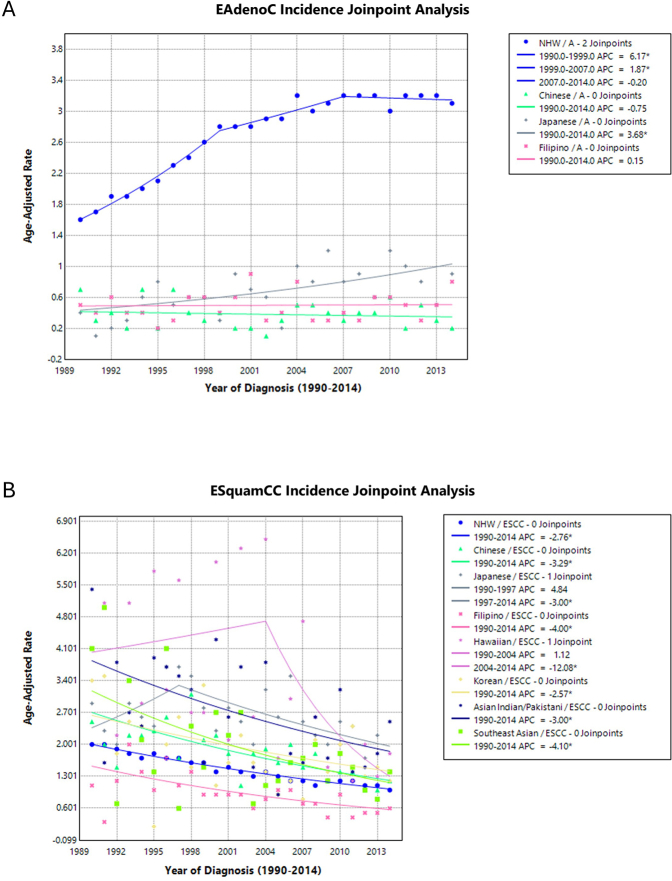


## Supplementary materials

Figure A1
