# Supplementary material for: Disparities in Gastrointestinal Cancer Incidence in Asian American, Native Hawaiian, and Other Pacific Islander Groups
Source: Gastro Hep Adv. 2025 Mar 15;4(6):100653. doi: 10.1016/j.gastha.2025.100653 (PMC12140049; doi:10.1016/j.gastha.2025.100653)
Supplement: Figure A1 [file mmc1.docx]

| **Cancer site** | **Pathologic subtype** | **Histological Codes** |
| --- | --- | --- |
| Colorectal Cancer (CRC) | Adenocarcinoma | 8140-8147, 8210-8211, 8220-8221, 8260-8263, 8480-8481, 8490 |
| Hepatic Cancer (HCC) | Hepatocellular Carcinoma | 8170-8175 |
| Gastric Cancer (GC) | Adenocarcinoma | 8140-8147, 8210-8211, 8214, 8220-8221, 8230-8231, 8255, 8260-8263, 8310, 8480-8481, 8490, 8510, 8560-8562, 8570-8576 |
| Pancreatic Cancer (PC) | Adenocarcinoma | 8140, 8145, 8154, 8255, 8260, 8440-8441, 8460, 8480-8481, 8490, 8500, 8504, 8507, 8510, 8514, 8521, 8523, 8550-8551, 8560, 8570, 8574 |
| Esophageal Cancer (EC) | Adenocarcinoma (EAdenoC) | 8140-8141, 8143-8145, 8154, 8190, 8200-8202, 8210-8215, 8220-8221, 8230-8231, 8255, 8260-8263, 8310, 8401, 8480-8482, 8490, 8550-8551, 8570-8574, 8576 |
| Esophageal Cancer (EC) | Squamous Cell Carcinoma (ESquamCC) | 8050-8053, 8070-8078, 8082-8083 |

Supplemental Table 1. Histological codes included in each cancer site
